# Effect of induction therapy on the expression of molecular markers associated with rejection and tolerance

**DOI:** 10.1186/s12882-015-0141-2

**Published:** 2015-08-19

**Authors:** Eva Krepsova, Irena Tycova, Alena Sekerkova, Peter Wohlfahrt, Petra Hruba, Ilja Striz, Birgit Sawitzki, Ondrej Viklicky

**Affiliations:** Transplant Laboratory, Centre for Experimental Medicine, Institute for Clinical and Experimental Medicine, Prague, Czech Republic; Department of Clinical and Transplant Immunology, Institute for Clinical and Experimental Medicine, Prague, Czech Republic; Department of Preventive Cardiology, Cardiology Centre, Institute for Clinical and Experimental Medicine, Prague, Czech Republic; Institute of Medical Immunology and Berlin-Brandenburg Centre for Regenerative Therapies (BCRT), Charité University Medicine, Berlin, Germany; Department of Nephrology, Transplant Centre, Institute for Clinical and Experimental Medicine, Videnska 1958, 14021 Prague, Czech Republic; Biomedical Centre, Faculty of Medicine in Plzen, Charles University in Prague, Plzen, Czech Republic

**Keywords:** Basiliximab, *FOXP3*, Kidney transplantation, rATG, Rejection, Tolerance

## Abstract

**Background:**

Induction therapy can improve kidney transplantation (KTx) outcomes, but little is known about the mechanisms underlying its effects.

**Methods:**

The mRNA levels of T cell-related genes associated with tolerance or rejection (*CD247, GZMB, PRF1, FOXP3, MAN1A1, TCAIM,* and *TLR5*) and lymphocyte subpopulations were monitored prospectively in the peripheral blood of 60 kidney transplant recipients before and 7, 14, 21, 28, 60, 90 days, 6 months, and 12 months after KTx. Patients were treated with calcineurin inhibitor-based triple immunosuppression and induction with rabbit anti-thymocyte globulin (rATG, *n* = 24), basiliximab (*n* = 17), or without induction (no-induction, *n* = 19). A generalized linear mixed model with gamma distribution for repeated measures, adjusted for rejection, recipient/donor age and delayed graft function, was used for statistical analysis.

**Results:**

rATG treatment caused an intense reduction in all T cell type population and natural killer (NK) cells within 7 days, then a slow increase and repopulation was observed. This was also noticed in the expression levels of *CD247*, *FOXP3*, *GZMB*, and *PRF1.* The basiliximab group exhibited higher *CD247*, *GZMB*, *FOXP3* and *TCAIM* mRNA levels and regulatory T cell (Treg) counts than the no-induction group. The levels of *MAN1A1* and *TLR5* mRNA expressions were increased, whereas *TCAIM* decreased in the rATG group as compared with those in the no-induction group.

**Conclusion:**

The rATG induction therapy was associated with decreased T and NK cell-related transcript levels and with upregulation of two rejection-associated transcripts (*MAN1A1* and *TLR5*) shortly after KTx*.* Basiliximab treatment was associated with increased absolute number of Treg cells, and increased level of *FOXP3* and *TCAIM* expression.

## Summary

In a prospective open study, rATG induction therapy was associated with profound decreases of T and NK cell-related transcripts and with the upregulation of two rejection-associated transcripts (*MAN1A1A* and *TLR5*) in the early post-KTx period*.* Basiliximab induction was associated with increased absolute number of Treg cells, and increased expression of tolerance associated markers *FOXP3* and *TCAIM.*

## Background

Long-term allograft survival requires lifelong immunosuppression, the use of which could be accompanied by several side effects [1]. For many patients triple drug regimens are not necessary due to weak alloreactive responses. Therefore, a development of reliable tests that may help identify patients suitable for drug minimization or at risk for rejection is needed. Several markers of rejection or operational tolerance (defined as good and stable graft function in immunosuppression free patients) have been identified [2–8].

Granzyme B (*GZMB*) and perforin (*PRF1*) are effector molecules produced by cytotoxic T and natural killer (NK) cells. Perforin forms a pore in the target cell membrane and thus facilitates the entry of granzyme B and other compounds into the cell which subsequently leads to apoptosis [9]. The urine and peripheral blood transcript levels of *PRF1* and *GZMB* were shown to be increased in kidney transplant recipients with acute rejection [2, 3]. The ζ-chain of T cell receptor (CD247) is a part of T-cell receptor-CD3 complex on T cells and activating receptors on NK cells [10]. Transcription of *CD247* was shown to be downregulated in peripheral blood lymphocytes from patients with long-term surviving kidneys [4, 10]. Toll-like receptor 5 (TLR5) is a member of TLR family which plays a fundamental role in the pathogen recognition and associated activation of innate immunity. The expression of *TLR5* was downregulated in operationally tolerant kidney graft recipients [5]. FoxP3 (forkhead box P3) is a key transcription factor in CD4^+^CD25^+^FoxP3^+^ regulatory T cells (Tregs), necessary for their differentiation and maintenance in the periphery [11]. Peripheral blood mRNA levels of *FOXP3* were higher in patients with operational tolerance or stable kidney graft function compared to patients with chronic rejection [7, 8]. A reduced gene-expression ratio of *FOXP3* to α-1,2-mannosidase (*MAN1A1*) was observed for chronically rejecting patients [5, 6]. Alpha-1,2-mannosidases (*MAN1A1*) are transmembrane proteins that specifically cleave α-1,2-linked mannose residues from oligosaccharides and are involved in the synthesis and maturation of N-glycoproteins [12]. *TCAIM* (T cell activation inhibitor, mitochondrial; previously named *TOAG-1*) was highly expressed during the induction and maintenance of tolerance, but was downregulated during acute rejection [6].

The induction therapy (with either lymphocyte-depleting agents including rabbit anti-thymocyte globulin (rATG) or IL-2 receptor antagonist such as basiliximab) is recommended as part of the initial immunosuppressive regimen in kidney transplant recipients to reduce acute rejection and/or to allow the reduction of other components of the regimen [13].

We and others previously demonstrated that induction therapy with rATG was associated with the expansion of relative numbers of CD4^+^CD25^+^FoxP3^+^ Treg during T cell depletion [14, 15] and that basiliximab induction caused the transient appearance and disappearance of CD4^+^CD25^low/-^FoxP3^+^ and CD4^+^CD25^+^FoxP3^+^ Treg, respectively [14, 16]. Furthermore we showed that high ratios of CD4^+^FoxP3^+^ Tregs to effector T cells in peripheral blood of kidney graft recipients treated with basiliximab induction in early post-transplant period were associated with the absence of rejection [14].

However, little is known about the effect of rATG and basiliximab on the expression of genes associated with rejection or operational tolerance in clinical kidney transplantation. Therefore, in order to address this issue, we measured relative quantities of seven selected molecular markers associated with rejection or tolerance (*CD247, GZMB, PRF1, MAN1A1, TLR5, FOXP3* and *TCAIM*) and numbers of lymphocyte subpopulations (CD3^+^, CD4^+^, CD8^+^, NK, CD4^+^FOXP3^+^) in the peripheral blood of kidney transplant recipients treated with rATG, basiliximab or no-induction.

## Methods

### Patients and samples

Between September 2009 and November 2010, 75 consecutive recipients of kidney transplants from deceased donors were enrolled in a single-centre prospective study. Written informed consent was obtained from all participants. The study protocol was approved by the Ethics Committee of the Institute for Clinical and Experimental Medicine (No. 608-08-10). Sixty patients met all of the following inclusion criteria for the study: 1) sufficient mRNA obtained during at least 7/9 sampling time-points, 2) unchanged maintenance immunosuppression, and 3) no steroid-resistant rejection.

Patients were treated in accordance with the centre’s immunosuppression treatment protocol which consisted of triple maintenance therapy with a calcineurin inhibitor (CNI; tacrolimus or cyclosporine A), mycophenolate mofetil, and corticosteroids. Patients were recruited for a specific induction therapy on the basis of personal immunologic risk factors. Patients with a panel-reactive antibody (PRA) score ≥ 50 % or with previous renal transplantation received 1–1.5 mg/kg/day rATG (Thymoglobulin®, Genzyme Corporation, Cambridge, MA, *n* = 24) in 2–7 doses during the first week after KTx. Patients with PRA scores of 20–49 % or the ones who received a kidney from an extended criteria donor were treated with 20 mg of basiliximab (Simulect®, Novartis, Basel, Switzerland, *n* = 17) on the day of KTx and 4 days after. Patients with PRA score < 20 % received no induction therapy (*n* = 19).

Peripheral blood samples were collected before and 7, 14, 21, 28, 60, 90 days, 6 months, and 12 months after KTx. Except for differences in the retransplantation frequency, mean PRA score, and donor age, the clinical characteristics did not differ significantly among the three groups (Table 1).

**Table 1:** Demographic characteristics at the time of transplantation

Variable	No-induction	rATG	Basiliximab	*P* value
Number	19	24	17	
Gender (M/F)	9/10	16/8	9/8	ns^†^
Recipient age (years)*	57 [27; 70]	54 [21; 78]	53 [25; 65]	ns^‡^
Donor age (years)*	52 [16; 68]	46 [18; 74]	61 [21; 75]	<0.05^‡a^
HLA MM*	3 [2; 6]	3 [1; 5]	3 [2; 5]	ns^‡^
1^st^/2^nd^ and 3^rd^ KTx (n)	19/0	11/13^§^	16/1	<0.0001^†^
PRA (%)*	4 [0; 22]	68 [0; 96]	6 [2; 63]	<0.0001^‡b^
CNI (TAC/CsA) (n)	21/4	28/0	18/0	ns^†^
CIT (hours)*	16.2 [11.0; 20.7]	15.4 [7.7; 20.1]	17.2 [7.7; 21.0]	ns^‡^
Dialysis time (years)*	2.0 [0.2; 5.7]	1.9 [0.5; 6.4]	2.0 [0.6; 4.9]	ns^‡^
Cause of renal failure				ns^†^
Primary GN	9	9	4	
Hereditary diseases	2	5	4	
Diabetic or ischemic nephropathy	6	2	7	
TIN	2	1	1	
ANCA vasculitis or lupus nephritis	0	4	0	
Other causes	0	3	1	

*ANCA* Anti-neutrophil cytoplasmic antibodies, *CIT* cold ischemic time, *CNI* calcineurin inhibitor, *CsA* cyclosporine A, *GN* glomerulonephritis, *HLA MM* HLA mismatch, *PRA* historical panel-reactive antibodies, measured every 3 months before transplantation (the highest number in each patient was considered), *TAC* tacrolimus, *TIN* tubulointerstitial nephritis, *TxR* renal transplantation

*Median [min; max]; ^†^Chi square test *P* value; ^‡^Kruskal-Wallis test *P* value

Dunn's Multiple Comparison Test: ^a^Significant difference between the basiliximab group and the rATG group and a ^b^significant difference between rATG and the no-induction or basiliximab group

^§^2 patients had a 3^rd^ transplantation

### Histology and treatment of rejection

Kidney graft biopsies were performed on the basis of clinical indications (case biopsies) or 90 days after KTx, as defined by the protocol. Acute rejection was diagnosed according to the Banff’05 classification [17]. Borderline changes and grade I or IIA T cell-mediated rejection were treated with 1.5–2 g of methylprednisolone. Antibody-mediated rejection was treated by plasma exchange and intravenous immunoglobulin alternately over the 10-day period.

### Flow cytometry and isolation of peripheral blood mononuclear cells

Venous blood samples were collected into sterile EDTA-containing tubes. Lymphocytes from peripheral blood (100 μL; ~1 × 10^6^ cells) were labelled with a 4-color monoclonal antibody (mAb) panel: CYTO-STAT tetraChrome CD45-FITC (clone: B3821F4A)/CD56-RD1 (clone: N901/NKH1)/CD19-ECD (clone: J3-119)/CD3-PC5 (clone: UCHT1) + CD16-PE (clone: 3G8) and CD45-FITC (clone: B3821F4A)/CD4-RD1 (clone: SFCI12T4D11)/CD8-ECD (clone: SFCI21Thy2D3)/CD3 (clone: UCHT1) (all Beckman Coulter, Brea, CA).

Extracellular staining of freshly prepared and isolated peripheral blood mononuclear cells was performed with anti-CD4-FITC (clone: RPA-T4) and anti-CD25-APC (clone: BC96) antibodies prior to intracellular staining with anti-FoxP3-PE (clone: PCH101). Tregs were stained for intracellular FoxP3 with the Human Regulatory T Cell Staining Kit (eBioscience, San Diego, CA, USA). An appropriate isotype control mAb (rat IgG2a-PE, cocktail of FITC and APC mouse IgG1) was used to establish the settings for FoxP3^+^ Treg analysis.

Stained samples were analysed in the FC 500 flow cytometer with CxP and Kaluza software (Beckman Coulter). Flow cytometric analyses were performed with at least 100 gated events. Lymphocyte subpopulations were defined as follows: T lymphocytes, CD45^+^CD3^+^; cytotoxic T lymphocytes, CD45^+^CD3^+^CD8^+^; and NK cells, CD45^+^CD3^−^CD16^+^CD56^+/-^. Because basiliximab may downregulate CD25 [16, 18] or interfere with some anti-CD25 mAbs used for flow cytometry [19], Tregs were defined as CD3^+^CD4^+^FoxP3^+^.

### Gene expression analysis and RNA isolation

Peripheral blood was drawn directly into PAXgene tubes (Qiagen, Hilden, Germany), frozen, and stored at -20 °C until analysis. Whole-blood RNA was extracted with the PAXgene Blood RNA Kit with DNAse I treatment (Qiagen). The purity and concentration of the RNA were assessed in an ultraviolet–visible spectrophotometer (NanoDrop 2000, Thermo Scientific). The RNA isolation method routinely used in our laboratory was validated and standardized on reference samples, to eliminate errors and ensure the same standards across all measurements. The quality of RNA samples obtained by the standard isolation protocol was assessed with the Agilent 2100 Bioanalyzer (Agilent Technologies). An RNA integrity number of 8–10 indicated high-quality RNA suitable for further analysis.

### Quantitative RT-PCR analysis

SuperScript™ II Reverse Transcriptase (Invitrogen, Carlsbad, CA, USA) was used to synthesize cDNA from 2 μg of total RNA isolated from blood samples. Seven genes were selected on the basis of them being previously described as being associated with rejection or tolerance: *GZMB*, *PRF1*, *CD247*, *FOXP3*, *TCAIM* (*C3orf23*), *MAN1A1*, and *TLR5*. Gene expression profiles of these seven genes were determined by quantitative real-time RT-PCR (qRT-PCR, 2-^ΔΔCt^ method), using *HPRT1* and *PGK1* as reference genes. The cDNA from one control blood sample was used for calibration. The mRNA levels were quantified in triplicate for each sample with a predesigned TaqMan® Gene Expression Assay (Hs01554355_m1 for *GZMB* [granzyme B], Hs00169473_m1 for *PRF1* [perforin 1], Hs00167901_m1 for *CD247*, Hs00203958_m1 for *FOXP3*, Hs00603313_m1 for *C3orf23* [TOAG or TCAIM], Hs00195458_m1 for *MAN1A1* [α-1,2-mannosidase], and Hs00152825_m1 for *TLR5*) and the TaqMan® Fast Advanced Master Mix (Applied Biosystems). Quantitative RT-PCR amplification was performed on an ABI Prism® 7900 H.T. Sequence Detection system (Applied Biosystems). Relative quantification analysis was performed in 96-well plates, using the RQ Manager 1.2. software for automated data analysis (Applied Biosystems).

### Statistical analyses

Characteristics of the rATG, basiliximab, and no-induction groups were compared by the Kruskal-Wallis test for continuous variables or the *χ*^2^ test for categorical variables. Data were expressed as the median [min; max] or as absolute numbers (*n*). A generalized linear mixed model for repeated measures, adjusted for rejection, donor/recipient age, and incidence of delayed graft function, was used for testing differences in the peripheral blood gene expression and the absolute and relative numbers of lymphocyte subpopulations between groups. Due to the non-normal distribution of data with long right tails, the dependent variable was subjected to gamma regression, with the data expressed as estimated marginal means ± SEM. Calculations were done with SPSS 20 (IBM Corporation, Somers, NY) and GraphPad Prism 5 (GraphPad Software, La Jolla, CA). A two-sided P-value ≤ 0.05 was considered statistically significant.

## Results

### Patient survival, graft function, and rejection

One of the 60 patients died during the follow-up period (day 223 after KTx) due to acute myocardial infarction. There were no differences among the groups in terms of delayed graft function (no-induction: 4/19, 21 %; rATG: 6/24, 25 %; basiliximab: 7/17, 41 %) or the 12-month serum creatinine, eGFR, and proteinuria results (Table 2). T cell-mediated rejection occurred within 12 months after KTx in 2/19 (10.5 %) of patients without induction and in 3/17 (17.6 %) of patients treated with basiliximab. Antibody-mediated rejection developed in 4/24 (16.7 %) of patients treated with rATG during follow-up. Borderline changes occurred in 7/19 (36.8 %) of patients from no-induction group, 4/24 (16.7 %) of patients from the rATG group, and 7/17 (41.2 %) of patients from the basiliximab group.

**Table 2:** Graft function at 12 months post transplantation

Variable	No-induction	rATG	Basiliximab	*P* value
SCr (μmol/L)*	120[73; 260]	121 [51; 261]	137 [82; 246]	ns^‡^
eGFR (mL/s/1.73 m^2^)*	0.80 [0.28; 1.18]	0.89 [0.38; 1.81]	0.65 [0.34; 1.45]	ns^‡^
Proteinuria (g/24)*	0.18 [0.07; 2.58]	0.18 [0.07; 11.58]	0.25 [0.08; 1.11]	ns^‡^

*eGFR* estimated glomerular filtration rate, *SCr* serum creatinine

*Median [min; max]; ^‡^Kruskal-Wallis test *P* value

### T and NK cells

**Fig. 1:**
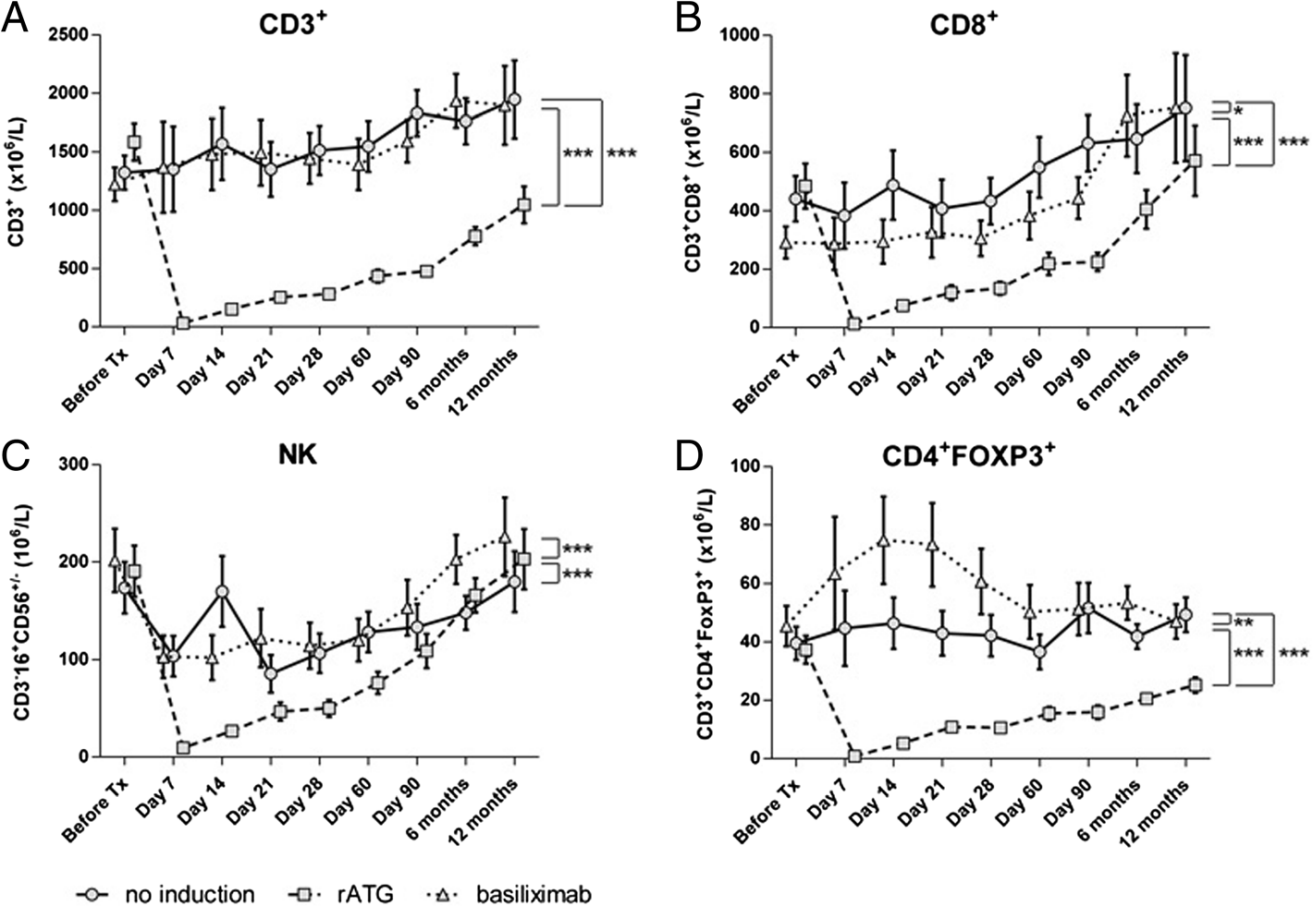
Effect of different inductive agents on the absolute numbers of T and NK cells in the peripheral blood of kidney transplant recipients during a 1-year follow-up period. Absolute numbers of T cells (**a**), CD8^+^ T cells (**b**), NK cells (**c**), and CD4^+^FOXP3^+^ Tregs (**d**) measured by flow cytometry in the peripheral blood from patients treated with rATG (*squares on dashed line*), basiliximab (*triangles on dotted line*), or without induction (*circles on solid line*). Data are presented as estimated marginal means ± SEM

In the rATG group, depletion of T cells (CD3^+^), the subpopulations (CD4^+^ T cells, CD8^+^ T cells, and CD4^+^FoxP3^+^ Tregs), and NK cells were observed at 7 days post-KTx, followed by a slow repopulation (Fig. 1a–d; data for CD4^+^ not shown). The CD8^+^ T and NK cell counts reached or exceeded their pre-KTx values at 12 month. The total cell counts for CD3^+^ T cells, CD4^+^ T cells, and CD4^+^FoxP3^+^ Tregs, however, only reached 66, 34, and 68 % of the preKTx values respectively, at 12 month. The total CD3^+^ and CD8^+^ T cells reached substantially higher counts at 12 month as compared to the pre-KTx values in the no-induction and basiliximab groups (CD3^+^: 147 % and 155 %, CD8^+^: 170 % and 257 %, respectively).

The absolute numbers of T cells, the subpopulations (CD4^+^, CD8^+^, and CD4^+^FoxP3^+^ Tregs), and NK cells were lower in the rATG group compared to the no-induction and basiliximab groups during follow-up (all P < 0.001; Fig. 1a–d; data for CD4^+^ not shown). CD8^+^ T cells were decreased and the Treg cells were increased in the basiliximab group as compared to the no-induction group (*P* < 0.05 and *P* < 0.01; Fig. 1b, d). In the basiliximab group, the number of Tregs increased soon after KTx, peaking on day 14 and then decreasing towards the pre-KTx level (Fig. 1d).

In contrast to the absolute numbers, the frequency of CD4^+^FoxP3^+^ Tregs among the CD4^+^ T cells was increased compared to the pre-KTx levels at day 7 in the rATG and basiliximab group. In rATG group the Treg frequency peaked at day 21 (to 303 % of the pre-KTx value) and remained high (194 % of the pre-KTx value) at the end of the follow-up period; in basiliximab group CD4^+^FoxP3^+^ Treg frequencies decreased towards the pre-KTx levels after day 7 and remained lower compared to rATG group (*P* < 0.001; data for Treg frequencies not shown). In the no-induction group, the Treg frequencies were stable and lower than those of the rATG and basiliximab group throughout the follow-up period (*P* < 0.001 and *P* < 0.05, respectively; data for Treg frequencies not shown).

### Rejection-associated transcripts

**Fig. 2:**
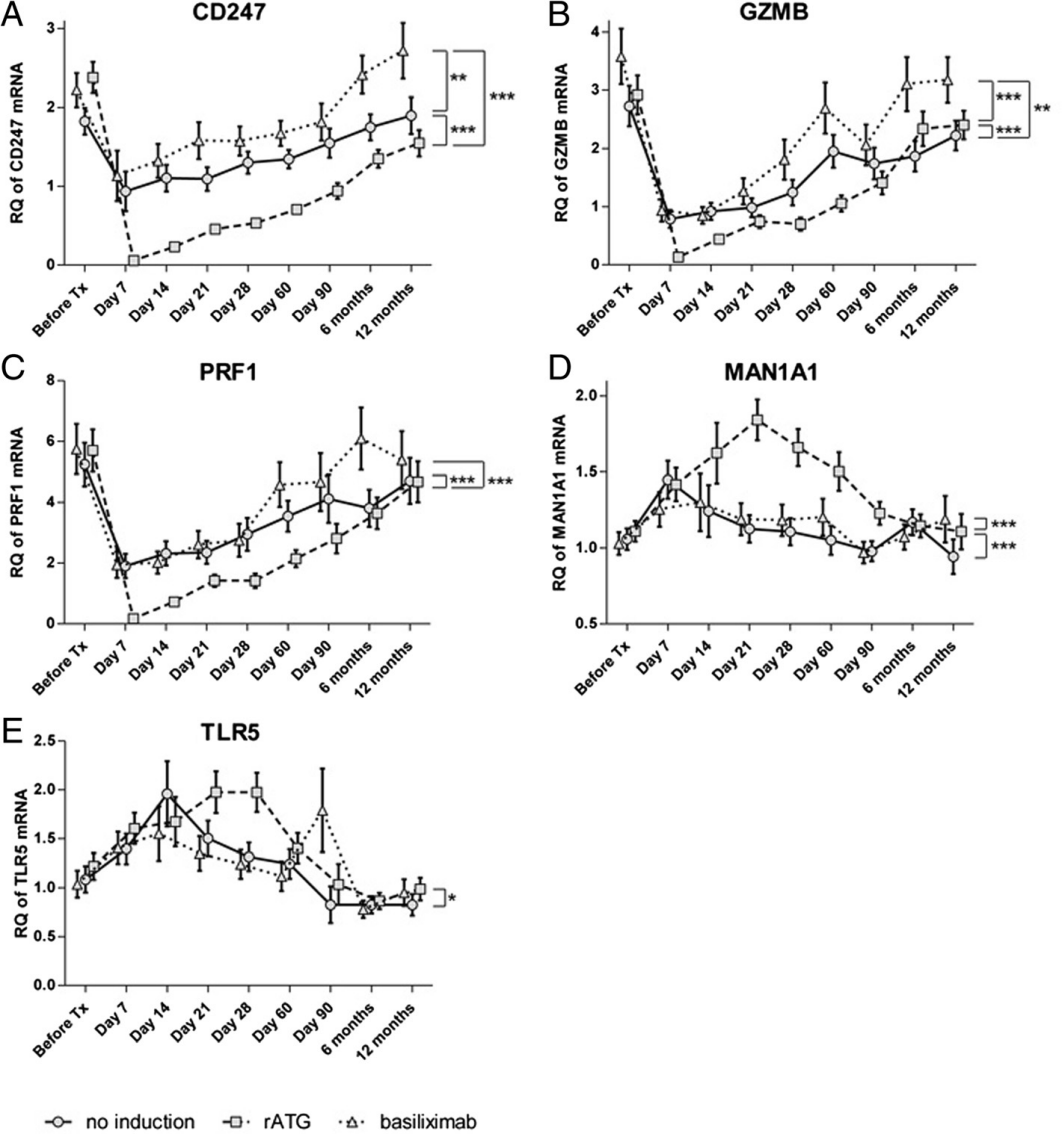
Effect of different inductive agents on expressions of rejection-associated transcripts expressed in the peripheral blood of kidney transplant recipients during a 1-year follow-up period. Relative quantity (RQ) of *CD247* (**a**)*, GZMB* (**b**), *PRF1* (**c**), *MAN1A1* (**d**), or *TLR5* (**e**) mRNA measured by qRT*-*PCR in peripheral blood from patients treated with rATG (*squares on dashed line*), basiliximab (*triangles on dotted line*), or without induction (*circles on solid line*). Data are presented as estimated marginal means ± SEM

In all groups, transcripts for *CD247*, *GZMB*, and *PRF1* decreased on day 7, thereafter increasing slowly towards their pre-KTx levels. The transcript levels decreased to zero and were lowest among the groups (all *P* < 0.001; Fig. 2a–c) in rATG group. Higher *CD247* and *GZMB* expression levels (both *P* < 0.01) were found in the basiliximab group compared to the no-induction group (Fig. 2a, b).

*MAN1A1* expression was highest (*P* < 0.001) in the rATG group as compared to basiliximab and no-induction groups (Fig. 2d). In all groups, the *MAN1A1* expression was increased on day 7 compared to the pre-KTx level. *MAN1A1* expression continued to increase in the rATG group, whereas it stabilized in the other two groups on day 14. In the rATG group, the *MAN1A1* expression peaked at day 21 and, thereafter, decreased towards its pre-KTx value. Substantial differences in *MAN1A1* expression were observed between the rATG group and other groups from days 21 to 90 (Fig. 2d). The *TLR5* mRNA expression was higher in the rATG group than in the no-induction group (*P* < 0.05; Fig. 2e).

### Tolerance-associated transcripts

**Fig. 3:**
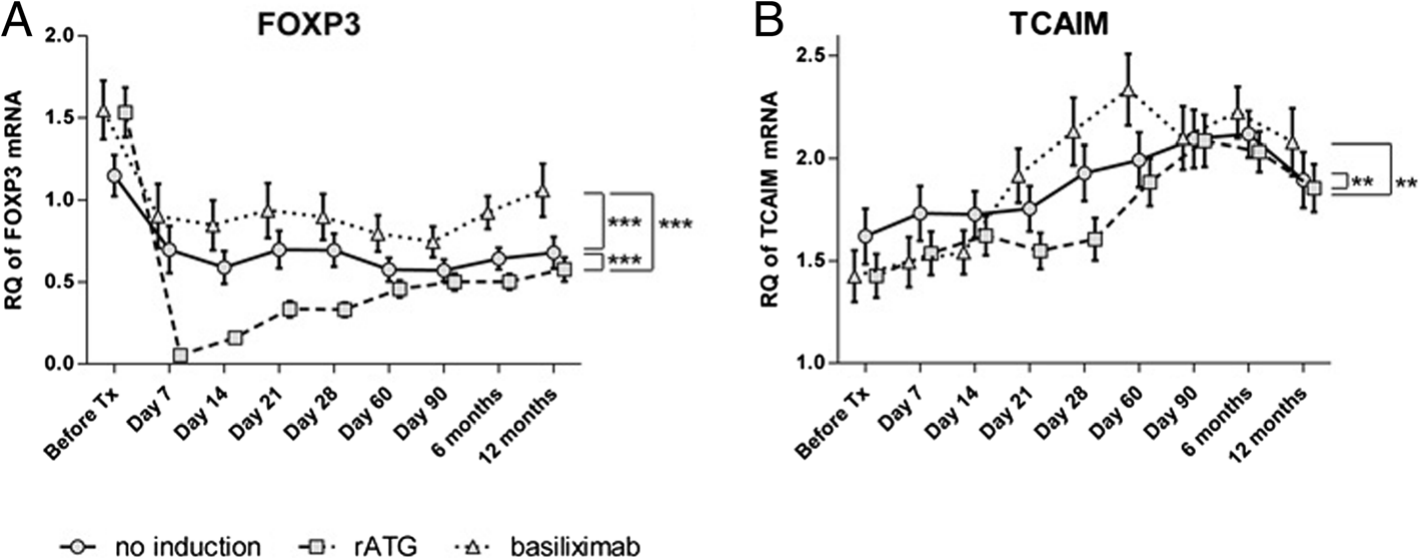
Effect of different inductive agents on tolerance-associated transcripts expressed in peripheral blood of kidney transplant recipients during a 1-year follow-up period. Relative quantity (RQ) of *FOXP3* (**a**) or *TCAIM* (**b**) measured by qRT*-*PCR in peripheral blood from patients treated with rATG (*squares on dashed line*), basiliximab (*triangles on dotted line*), or without induction (*circles on solid line*). Data are presented as estimated marginal means ± SEM

Similar to the findings for *CD247*, *GZMB*, and *PRF1*, the mRNA expression of *FOXP3* was decreased in all groups on day 7 compared to the pre-KTx level with the most profound decrease and subsequent increase towards the pre-KTx level in the rATG group. *FOXP3* expression was lowest in the rATG group; it was higher in the basiliximab group than in the no-induction group (all *P* < 0.001; Fig. 3a).

The trend of the mRNA expression ratio of *FOXP3* to *MAN1A1* was similar to that of the *FOXP3* expression; the ratio decreased on day 7 in all groups, with the most profound decrease and subsequent increase towards pre-KTx levels being seen in the rATG group. The lowest ratio was found in the rATG group (all *P* < 0.001; data not shown). The *TCAIM* mRNA expression constantly increased in all groups, with basiliximab- and rATG-treated patients displaying the fastest and slowest increases, respectively, among the groups (*P* < 0.01; Fig. 3b).

## Discussion

To the best of our knowledge, this is the first prospective trial that aimed to evaluate the effects of different induction agents on molecular markers associated with rejection or operational tolerance in kidney transplant recipients. We analysed the relative quantity of seven selected T cell-related transcripts, associated with operational tolerance or rejection, and lymphocyte subpopulations in KTx patients treated with different induction regimens. In our study the rATG induction therapy was associated with profound decrease of T and NK cells, as well as T cell transcripts that are exclusively expressed by these cell types. In rATG group the transient upregulation of *MAN1A1* and *TLR5* transcripts, previously shown to be associated with chronic rejection [5], was observed. Basiliximab induction resulted in a transient increase in CD4^+^FoxP3^+^ Tregs, accompanied by the highest peripheral expression levels of markers associated with operational tolerance (*FOXP3* and *TCAIM*).

Many studies have documented the dose-dependent depletional effect of rATG on T and NK cells [20–24]. However, little is known about transcript-level changes due to rATG treatment. Simon *et al.* described the effect of ATG induction therapy on expression of 10 immunologically relevant genes in the early post-transplant period and found decrease of *GZMB* and *PRF1* expression [25]. We observed a profound drop in the relative mRNA quantities of *CD247*, *GZMB*, *PRF1*, and *FOXP3* early after transplantation in rATG group. These transcripts are expressed exclusively by T and NK cells and are present at different levels in rejecting or operationally tolerant patients [2, 3, 7, 8]. The profound drop was followed by a slow increase towards pre-KTx levels in rATG patients, corresponding to the depletion and progressive repopulation of the aforementioned cell types.

Interestingly, in the early period after KTx, there was a decrease in gene expression of *CD247*, *GZMB*, *PRF1*, and *FOXP3*, followed by a return to pre-KTx levels, in patients who were treated with basiliximab or without induction. This finding can likely be explained by the decreased NK cell counts and the effect of maintenance therapy on gene expression. The calcineurin-dependent mechanism of action involves the binding of CNIs to their respective immunophilins. CNIs inhibit the transcription of proinflammatory and T cell-recruiting cytokines, such as interleukin (IL)-2 [26]. IL-2 regulates the gene expression of perforin and granzyme [27]. It directly promotes the transcription of FoxP3 [28, 29]. Moreover, IL-2 has been shown to augment TCR-ζ (CD247) expression in chronic inflammation [30].

While the expressions of *CD247*, *GZMB*, *PRF1*, and *FOXP3* were downregulated*, MAN1A1, TLR5* and *TCAIM* transcripts were upregulated early after KTx in all groups. These transcripts might be expressed preferentially by other cell types than T cells which are more susceptible to induction and maintenance immunosuppression. *TCAIM, MAN1A1* and *TLR5* are expressed not only by T cells, but also by macrophages/monocytes and dendritic cells [6, 31–35]. The Indices of Tolerance Research Network ranked *TLR5* as one of the top-10 gene markers for distinguishing between drug-free operationally tolerant and chronically rejecting kidney recipients. Specifically, *TLR5* was highly expressed in patients with chronic rejection, whereas it was downregulated in tolerant patients [5]. Similarly, increased expression of the other member of toll-like receptors *TLR4* was observed in kidney transplant recipients with chronic rejection compared to operationally tolerant patients [36]. After haematopoietic stem cell transplantation, patients experiencing graft-versus-host disease had increased peripheral *TLR5* expression; adoptive Treg therapy reduced this expression by preventing the disease [34]. Although the differences in *TLR5* expression in our study were statistically significant, the clinical significance remains questionable.

*TCAIM* has been shown to be highly expressed during the induction and maintenance of operational tolerance to donor alloantigens *in vivo*, resulting in the acceptance of kidney and heart allografts in rats and mice [6]. *TCAIM* expression was downregulated in the peripheral blood and the graft before rejection. This downregulation has been shown to occur in graft-infiltrating cells and after T-cell activation *in vitro* [6, 37]. The TCAIM protein is localized exclusively within mitochondria. TCAIM-expressing murine T cells have been shown to be more susceptible to apoptosis [38]. An increased *TCAIM* mRNA level was described after administration of regulatory macrophages to patients before living-donor KTx [39]. In a rat KTx model, intragraft expression of *TCAIM* was dramatically reduced in untreated rejecting recipients and in recipients who received an adoptive transfer of memory T cells [40]. Recently, it was shown that *TCAIM* inhibited spontaneous development of memory and effector T cells [41]. Higher *FOXP3* and *TCAIM* expressions along with Treg numbers in basiliximab group may suggest a protective potential of basiliximab. Here we showed that basiliximab induction was associated with a transient increase in absolute numbers of CD4^+^FoxP3^+^ Tregs which was in line with findings of Bluestone et al. [42]. However the debate has not been finished yet and there are also other observations [43, 44]. The rATG induction resulted in long-lasting and profound depletion of absolute numbers of CD4^+^FoxP3^+^ Tregs that corresponded to decreased level of *FOXP3* gene expression.

## Conclusions

Induction immunosuppression with rATG was associated with long-lasting suppression of T and NK cell-associated genes, and with the upregulation of markers associated with rejection, *MAN1A1* and *TLR5,* within the first month after KTx. In contrast, basiliximab induction resulted in a fast increase in CD4^+^FoxP3^+^ Tregs and expression of some markers associated with operational tolerance.

## Abbreviations

KTx: Kidney transplantation
rATG: rabbit anti-thymocyte globulin
NK: Natural killer cells
Treg: Regulatory T cell
GZMB: Granzyme B
PRF1: Perforin
CD247: ζ-chain of T cell receptor
FoxP3: Forkhead box P3
TLR5: Toll-like receptor 5
MAN1A1: α-1,2-mannosidase
TCAIM: T cell activation inhibitor, mitochondrial
CNI: Calcineurin inhibitor
